# Thioredoxin system: mechanisms and therapeutic potential in mental disorders

**DOI:** 10.3389/fphar.2026.1928981

**Published:** 2026-09-29

**Authors:** Le Zhang, Xin Wang, Ningning Guo, Jie Bai

**Affiliations:** 1 Faculty of Life Science and Technology, Kunming University of Science and Technology, Kunming, China; 2 Medical School, Kunming University of Science and Technology, Kunming, China; 3 Department of Radiation Therapy, The First Affiliated Hospital of Harbin Medical University, Harbin, China; 4 School of Mental Health, Jining Medical University, Jining, China

**Keywords:** mental disorders, neurogenesis, neuroinflammation, neurotransmitters, thioredoxin system

## Abstract

Mental disorders, including depression, schizophrenia (SCZ), autism spectrum disorders (ASD), and substance use disorders (SUD), impose a substantial global disease burden. The pathogenesis of these diseases is complex and not fully understood, which limits the therapeutic efficacy of existing interventions. Cumulative evidence indicates that redox imbalance, impaired neurogenesis, neuroinflammation, neurotransmitter dysregulation, mitochondrial dysfunction, synaptic dysfunction, and ferroptosis represent core pathological processes underlying mental disorders. The thioredoxin (Trx) system, a core machinery for intracellular redox homeostasis, comprises Trx, thioredoxin reductase (TrxR), nicotinamide adenine dinucleotide phosphate (NADPH), and thioredoxin-interacting protein (TXNIP). Multiple studies have documented the Trx system dysregulation across diverse mental disorders contributes to disease onset and progression. In this review, we summarize the structural composition and biological functions of the Trx system and discuss its possible molecular mechanisms in four major mental disorders. We also describe crosstalk between the Trx system and the glutathione (GSH) system and dissect the therapeutic prospects and translational bottlenecks of Trx-targeted pharmacological strategies. Future research directions are also outlined. Collectively, the Trx system serves as a promising candidate target for unraveling redox-dependent pathophysiological mechanisms in mental disorders. Nevertheless, multi-dimensional mechanistic and translational investigations are urgently required before Trx-based therapeutics can yield tangible clinical benefits for patients with psychiatric conditions.

## 1 Introduction

Redox reactions are indispensable for sustaining normal physiological functions and stabilizing the microenvironment of biological macromolecules. Intracellular redox homeostasis refers to the dynamic equilibrium between oxidative and antioxidant systems and constitutes a fundamental cellular defense mechanism (Yang et al., 2022). Reactive oxygen species (ROS) and reactive nitrogen species (RNS), unavoidable by-products of cellular respiration and energy metabolism, exert both physiological and pathological effects (Valko et al., 2007). At moderate concentrations, ROS and RNS act as second messengers to modulate gene expression. By contrast, sustained excess of free radicals triggers oxidative damage to DNA, RNA, proteins, and membrane lipids (Valko et al., 2007). In humans, the brain accounts for about 2% of total body weight yet consumes roughly 20% of the body’s glucose supply (Mergenthaler et al., 2013). Enriched in oxidizable polyunsaturated fatty acids and redox-active metals, brain tissue renders the central nervous system (CNS) particularly vulnerable to oxidative stress (OS)-associated injury. Persistent OS disrupts neuronal structure and function, and ultimately drives the development of neurological and psychiatric disorders (Barron et al., 2017; Fraguas et al., 2019; Ramli et al., 2020). To counteract oxidative insults, the brain relies on multiple endogenous enzyme-based antioxidant defense systems. The thioredoxin (Trx) system is a highly conserved sulfhydryl-dependent antioxidant system composed of Trx, thioredoxin reductase (TrxR), thioredoxin-interacting protein (TXNIP), and nicotinamide adenine dinucleotide phosphate (NADPH) (Xue et al., 2022). Compelling evidence links Trx-system dysfunction tightly to mental disorders. Herein, we summarize the Trx system’s biological functions and molecular mechanisms and explore its therapeutic implications for mental disorders.

Literature was retrieved from PubMed and Web of Science, covering publications from January 2000 to June 2026. Search keywords included: Trx, Trx-1, Trx-2, TXNIP, TrxR, oxidative stress, neuroinflammation, ferroptosis, depression, schizophrenia (SCZ), autism spectrum disorder (ASD), substance use disorders (SUD), mental disorders, neurogenesis, mitochondrial dysfunction, immune injury. Inclusion criteria comprised peer-reviewed original animal and human studies, as well as systematic reviews and meta-analyses. Exclusion criteria were conference abstracts without full texts, unblinded single-case reports, and duplicate publications. To mitigate selection bias and improve reproducibility, we implemented a clear hierarchical framework for evidence prioritization, prioritizing human clinical trial data, standardized rodent *in vivo* behavioral research, and isolated *in vitro* molecular assays.

## 2 Trx system

### 2.1 Trx

Trx is a redox regulatory protein (12 kDa) with an amino acid sequence of -Cys-Gly-Pro-Cys- in the active center (Awan et al., 2022). Initially identified in *Escherichia coli*, Trx has since been found to be widely expressed across prokaryotes and eukaryotes. Structural analyses show that oxidized Trx has a central five-stranded β-sheet surrounded by four peripheral α-helix, with the dithiol/disulfide active site exposed on the protein surface (Collet and Messens, 2010). Three human Trx isoforms have been well characterized by their subcellular localization and tissue-expression profiles (Wang et al., 2022). Trx-1 is abundantly expressed in the cytoplasm. Under stress, Trx-1 translocates into the nucleus or traffics to the plasma membrane (Rhee et al., 2005; Monteiro et al., 2017), serving as the major isoform regulating redox reactions, transcription-factor activity, and apoptotic signal transduction. Trx-2 localizes predominantly to mitochondria (Lee et al., 2013) and plays a critical role in maintaining mitochondrial ROS homeostasis and mitochondrial-mediated apoptosis. Trx-3, also termed sperm-specific Trx, is exclusively expressed in the testis and participates in spermatogenesis (Lu and Holmgren, 2009). Under physiological conditions, reduced Trx-1 and Trx-2 directly bind and suppress the kinase activity of apoptosis signal-regulating kinase 1 (ASK-1), thereby blocking downstream apoptotic cascades (Fujino et al., 2006). Accordingly, modulation of the disulfide-bond status of Trx is essential for cell-survival signaling. Compared with Trx-2, Trx-1 contains three additional non-catalytic cysteine residues (Cys62, Cys69, and Cys73) (Ouyang et al., 2018). These residues can form alternative disulfide bonds, most notably the Cys62-Cys69 linkage (Zhang X. et al., 2015), indicating isoform-specific regulatory mechanisms. Given the high Trx-1 abundance in the brain and its direct engagement in neuronal signaling, the vast majority of psychiatric disorder-related studies focus on Trx-1 Figure 1.

**FIGURE 1:**
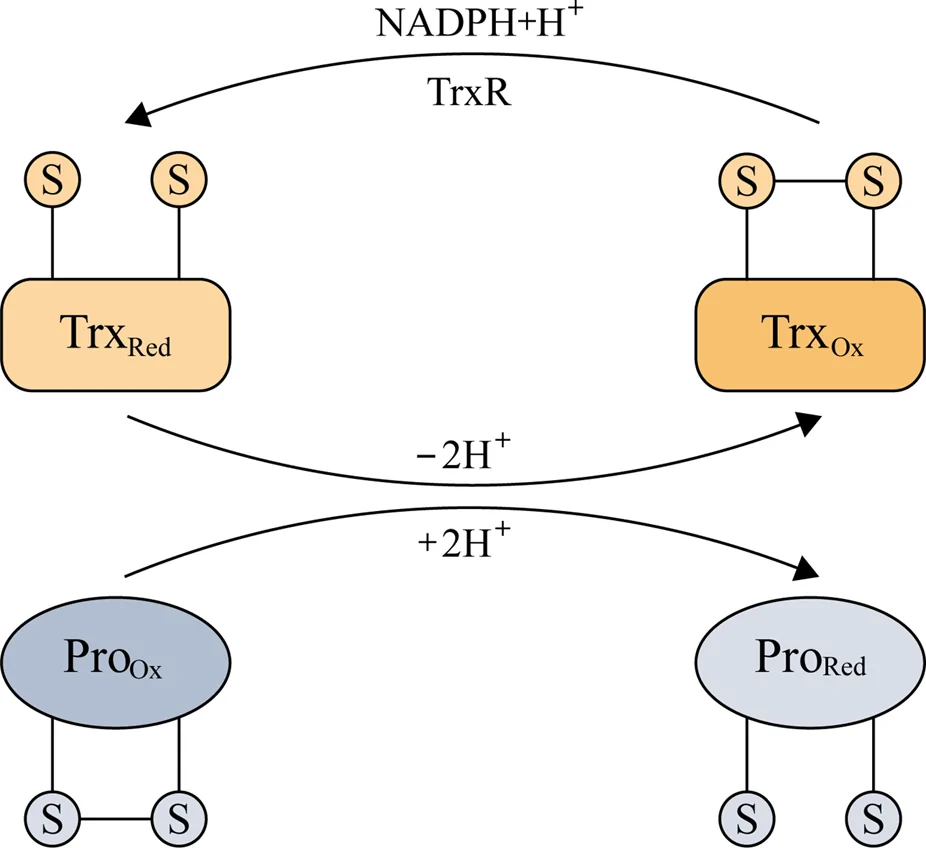
Conversion of oxidized Trx to reduced Trx. Reduced Trx is converted to oxidized Trx by transferring electrons to an oxidized protein. Oxidized Trx is converted to reduced Trx by obtaining electrons from NADPH under the action of NADPH-dependent TrxR. Red, reduced; Ox, oxidized; NADPH, nicotinamide adenine dinucleotide phosphate; TrxR, thioredoxin reductase.

### 2.2 TrxR

TrxR is a homodimeric protein containing selenocysteine, a flavoprotein of pyridine nucleotide-disulfide oxidoreductase (Lu and Holmgren, 2014; Nasoohi et al., 2018). It represents the only known enzyme capable of maintaining Trx in its reduced state under physiological conditions (Bjorklund et al., 2021). Three mammalian TrxR isoforms have been identified: TrxR1, TrxR2, and TrxR3 (Dipol et al., 2025). *In vitro* recombinant-protein assays demonstrate that wild-type (WT) TrxR bearing the selenocysteine (Sec) residue exhibits approximately 100-fold higher catalytic efficiency toward certain substrates (e.g., 5,5-dithiobis(2-nitrobenzoic acid)) relative to Sec-deficient variants (Zhong and Holmgren, 2000). Mammalian TrxR possesses an N-terminal redox motif (-Cys59-Val-Asn-Val-Gly-Cys64-), alongside a C-terminal redox center harboring cysteine and selenocysteine (-Gly-Cys497-Sec498-Gly-) (Zhong et al., 2000; Zhang B. et al., 2017). Functional TrxR exists as a homodimer. The N-terminal and C-terminal active centers of one TrxR subunit form disulfide-bond linkages with the C-terminal and N-terminal active centers of the partner subunit, respectively, generating two intermolecular disulfide bonds (Lee et al., 2013; Zhang J. et al., 2017). Using NADPH as the electron donor, the TrxR dimer reduces disulfide groups to dithiols. In other words, oxidized Trx is converted into its reduced form via thiol-disulfide exchange (Jia et al., 2019). Beyond Trx reduction, TrxR can also catalyze the reduction of hydrogen peroxide, lipid hydroperoxides, and other substrates.

In addition, the *Trx* promoter contains multiple stress-responsive cis-acting regulatory elements, including the antioxidant response element (ARE), oxidative stress response element (ORE), and cAMP response element (CRE) (Yoshihara et al., 2014). Constitutive *Trx-1* knockout causes embryonic lethality in mice, whereas *Trx-1* overexpressing transgenic mice display extended lifespans (Mitsui et al., 2002), indicating that Trx-1 is indispensable for fundamental biological processes. Upon stress, reduced Trx-1 translocates into the nucleus and enhances the DNA-binding affinity of transcription factors including nuclear factor kappaB (NF-κB) (Go et al., 2011) and p53. The Trx/TrxR axis is also broadly implicated in regulating cell differentiation, proliferation, and death signaling (Zhang B. et al., 2018).

### 2.3 TXNIP

TXNIP, an endogenous negative regulator of Trx, is an α-arrestin protein also known as vitamin D3-up-1 (VDUP1) or thioredoxin-binding protein-2 (TBP-2) (Nasoohi et al., 2018). It is encoded by the *TXNIP* gene located at human chromosome locus 1q21.1 (Zhou et al., 2011). The TXNIP protein consists of 391 amino acid residues and comprises mainly of inhibitory N-terminal and C-terminal domains, together with a C-terminal-tail PPxY motif. These unique inhibitory domains confer its primary function of Trx binding and suppression (Choi and Park, 2023). The cysteine residue Cys247 of TXNIP forms a mixed disulfide bond with Cys32 of reduced Trx to generate the TXNIP-Trx-1 complex that abrogates Trx-1 antioxidant activity (Patwari et al., 2006). TXNIP overexpression suppresses Trx-1 activity, elevates cellular oxidative stress, and inhibits cell proliferation. Interestingly, *TXNIP* knockout mice show barely altered basal Trx-1 activity under homeostatic conditions (Chutkow et al., 2008), which may be explained by TXNIP’s predominantly nuclear localization in unstressed cells.

Upon OS stimulation, TXNIP shuttles from the nucleus into the cytosol and mitochondria, where it binds to Trx-1 and Trx-2. This molecular interaction triggers the dissociation and activation of ASK-1, initiating ASK-1-mediated apoptosis (Lu and Holmgren, 2012). TXNIP also regulates inflammatory signaling. Activation of the NOD-like receptor pyrin domain containing 3 (NLRP3) inflammasome represents a central step in inflammatory responses (Abderrazak et al., 2015). The physical interaction between TXNIP and NLRP3 is a well-documented activation mechanism (Chen et al., 2008). NLRP3 belongs to the NLR protein family and is mainly composed of the nucleotide-binding and oligomerization (NACHT) domain, pyrin domain (PYD), and leucine-rich repeat (LRR) domain (Pan M. et al., 2022). Under resting conditions, intramolecular NACHT-LRR interactions maintain NLRP3 in an auto-inhibited conformation (Menu and Vince, 2011). When ROS levels rise, the inhibitory C-terminal domain of dissociated TXNIP binds to the LRR domain of NLRP3, relieving NLRP3 auto-inhibition (Zhou et al., 2010). Subsequently, NACHT-driven NLRP3 oligomerization occurs (Kelley et al., 2019), and the PYD domain recruits the PYD-containing ASC adaptor protein. The CARD domain of ASC further recruits pro-caspase-1 via CARD-CARD interaction, completing inflammasome assembly and activating caspase-1 (Wagner et al., 2018). Activated caspase-1 cleaves pro-IL-1β and pro-IL-18 to produce mature and secreted pro-inflammatory cytokines (Zhou et al., 2010; Shi et al., 2016). Additionally, TXNIP helps regulate cell cycle progression (Pan M. et al., 2022) and energy-metabolism pathways (Sullivan et al., 2018) Figure 2.

**FIGURE 2:**
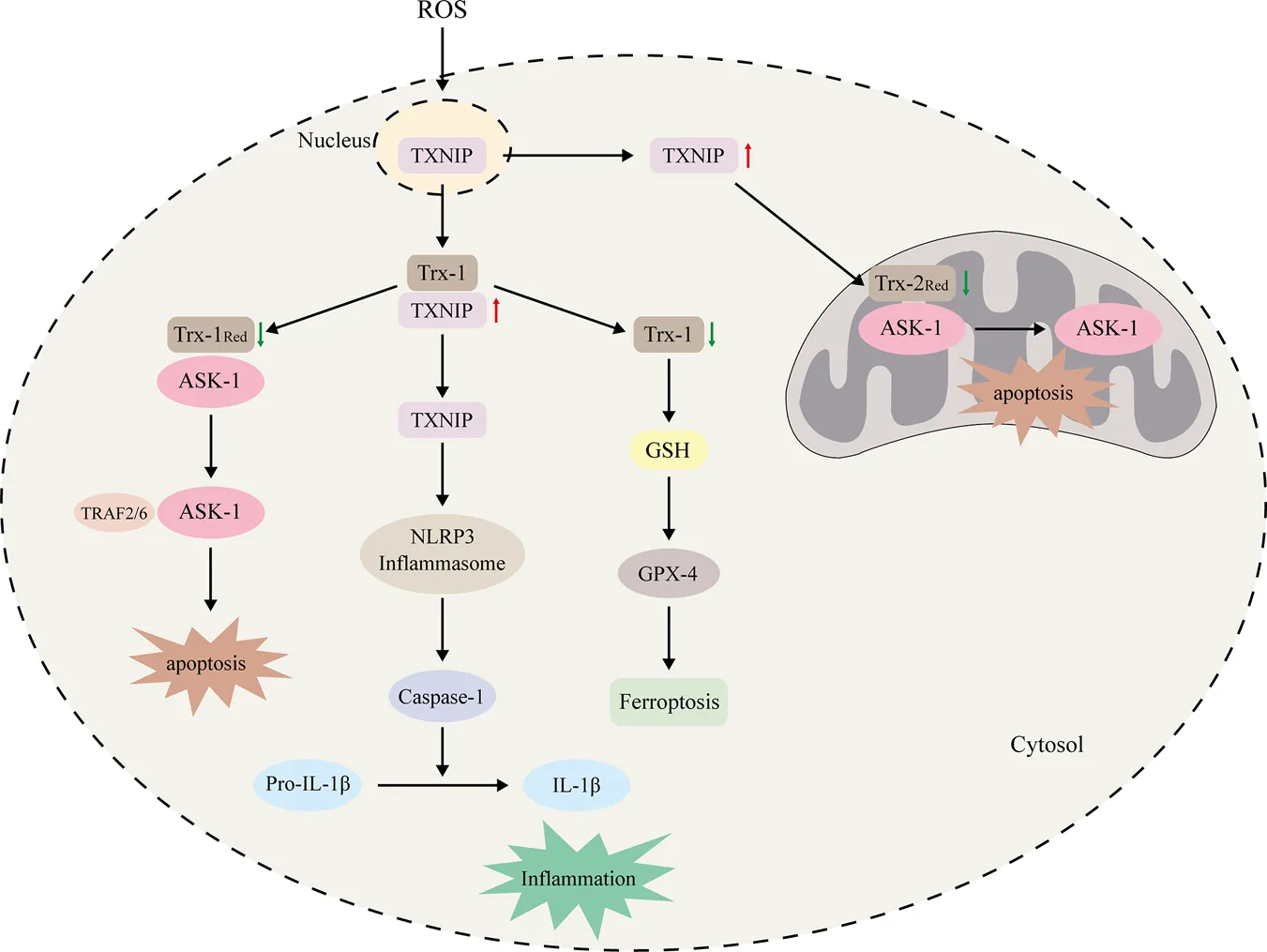
General functions of TXNIP. ASK-1, apoptosis signal-regulating kinase-1; GPX-4, glutathione peroxidase 4; NLRP3, NOD-like receptor thermal protein domain associated protein 3; ROS, reactive oxygen species; Trx-1, thioredoxin-1; TXNIP, thioredoxin interacting protein.

### 2.4 Crosstalk between the Trx system and the glutathione system

In addition to the Trx system, mammalian cells have another critical thiol-dependent antioxidant system: the glutathione (GSH) system (Lan et al., 2024). This system includes core components such as NADPH, glutathione reductase (GR), oxidized glutathione (GSSG), GSH, and glutaredoxin (Grx) (Lu et al., 2021). GR uses electrons donated by NADPH to reduce GSSG back to GSH, thereby maintaining an intracellular reductive microenvironment and reinforcing cellular antioxidant defense (Pannala et al., 2013). The Trx and GSH systems share NADPH derived from the pentose phosphate pathway as their primary source of reducing equivalents (Chen Z. et al., 2025), and both antioxidant cascades are transcriptionally governed by the nuclear factor erythroid 2-related factor 2 (Nrf2) -ARE axis (Gao et al., 2026). These two redox networks exhibit partial functional redundancy and compensatory crosstalk. Under specific cellular contexts, impairment of one antioxidant pathway can be partially buffered by the other to preserve basal redox homeostasis (Benhar, 2020). Nevertheless, distinct substrate specificities and functional divergences prevent full functional substitution between the two systems (Go et al., 2013). Although GSH system dysregulation has been more extensively characterized in psychiatric disorders (Mandal et al., 2022), accumulating evidence indicates that Trx system dysfunction exerts complementary and equally critical effects in mediating neuropsychiatric pathogenesis.

### 2.5 Trx system and pathological phenotype

The TXNIP/Trx complex serves as a crucial signaling hub that orchestrates CNS redox homeostasis. Under ROS accumulation, aberrantly elevated TXNIP destabilizes Trx-ASK-1 binding and triggers dissociation, launching the neuronal apoptotic cascade. Meanwhile, TXNIP facilitates NLRP3 inflammasome assembly and activation, provoking sustained, excessive neuroinflammatory responses in the CNS (Zhou et al., 2010). Moreover, Trx deficiency represses Nrf2 signaling and compromises overall cellular redox homeostasis (Sueblinvong et al., 2016). These interwoven molecular cascades give rise to diverse neuropathological insults, exemplified by amplified central inflammation (Bjørklund et al., 2022), perturbed central immune homeostasis, defective hippocampal neurogenesis (Tian et al., 2014), neurotransmitter dysregulation, impairments in synaptic structure and plasticity, mitochondrial dysfunction (Zaobornyj et al., 2019), and ferroptosis (Bai et al., 2021). Collectively, this multi-layered regulatory framework provides mechanistic insights for deciphering the molecular basis of psychiatric disorders.

## 3 Trx system and mental disorders

Mental disorders represent a global public-health challenge, characterized clinically by severe disturbances in an individual’s cognition, emotional regulation, or behavior (Shang et al., 2017). According to the latest statistics from the World Health Organization, nearly one-seventh of the global population is affected by mental disorders, and this prevalence is on the rise (World Health Organization, 2025a). Representative disease entities include depression, SCZ, ASD, and SUD (Huang et al., 2019). Accumulating evidence suggests disrupted equilibrium between oxidant and antioxidant defense systems in the brains of patients with mental disorders (Bell et al., 2024; Zhang et al., 2025), resulting in excessive OS. As a crucial endogenous regulatory module for maintaining intracellular redox homeostasis (Yang et al., 2024), the Trx system plays a vital role in the pathogenesis of multiple mental disorders (Table 1) (Figure 3). In the following sections, we elaborate on the Trx system’s roles and underlying mechanisms in mental disorders.

**TABLE 1:** Alterations of Trx system components in mental disorder-related human subjects and experimental models.

Mental disorders	Subjects	Model	Expression of Trx system	References
Depression	Human	Depression	TXNIP ↑ in the anterior cingulate cortex	(Zhou et al., 2018)
	Human	TRD	Plasma Trx-1 unchanged	(Aydın et al., 2018)
	Rat	CUMS	Trx-1 ↓, TXNIP ↑ in the hippocampus	(Song et al., 2018)
	Mice	LPS	Trx-1 ↓, TXNIP ↑ in the hippocampus	(Dang et al., 2019; Jing et al., 2019)
	Mice	Restraint stress	Trx-1 ↓ in the hippocampal	(Kim and Leem, 2019)
	Mice	CUMS	TXNIP ↑ in the hippocampus	(Su et al., 2017a)
	Mice	LPS/TNF-α	TXNIP ↑ in the hippocampus	(Camargo et al., 2021)
	Rat	Corticosterone	mRNA and protein level of TXNIP ↑ in the frontal cortex	(Pan et al., 2022b; Pan et al., 2022c)
	Mice	CUS	TXNIP ↑ in the frontal cortex and hippocampus	(Zhou et al., 2019)
	Mice	Streptozotocin	TXNIP ↑ in the hippocampus	(Su et al., 2023)
SCZ	Human	First-episode and long-term SCZ patient	Serum Trx-1 ↑	(Zhang et al., 2009)
	Mice	MK-801	TXNIP ↑ in the PFC	(Xue et al., 2022)
ASD	Human	ASD	Serum Trx-1 ↑	(Zhang et al., 2015a)
	Human	ASD	TrxR1 ↑ in neutrophils	(Alshehri et al., 2023a)
	Rat	VPA	TXNIP ↑ in the PFC	(Zhao et al., 2022)
	Mice	Shank3 KO	Trx1↓, TrxR↓, and Trx2↓in the Cortex	(Bazbaz et al., 2024)
SUD	Mice	Morphine-CPP	Trx-1↑ in the VTA, NAc, DG	(Li et al., 2018)
	Mice	Morphine-CPP reinstatement	Trx-1↑ in the NAc and hippocampus	(Guo et al., 2018)
	Mice	METH-CPP	Trx-1↓ in the VTA and NAc	(Huang et al., 2018b)
	Mice	METH-CPP reinstatement	Trx-1 ↑ in the NAc	(Huang et al., 2018c)
	Mice	METH-CPP extinction	Trx-1 ↓ in the VTA	(Huang et al., 2018a)

CUMS, chronic mild unpredictable stress; LPS, lipopolysaccharide; CUS, chronic unpredictable stress; Trx, thioredoxin; TXNIP, thioredoxin interacting protein; TRD, treatment-resistant depression; METH, methamphetamine; CPP, conditioned place preference; VTA, ventral tegmental area; NAc, nucleus accumbens; DG, dentate gyrus; SCZ, schizophrenia; PFC, prefrontal cortex; ↓, decreased; ↑, increased.

**FIGURE 3:**
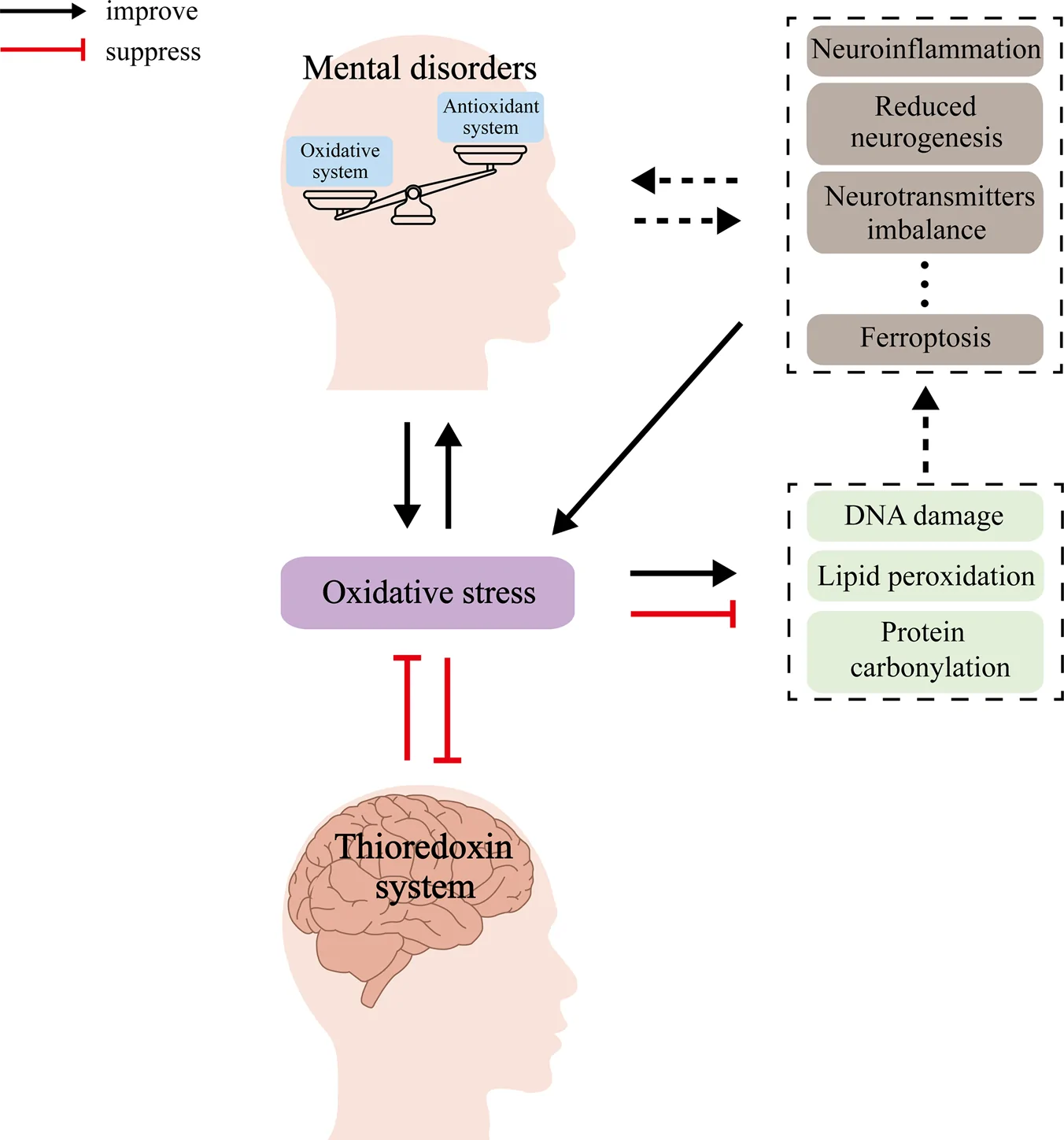
Schematic illustration of the Trx system-mediated redox regulation in the pathogenesis of mental disorders. The Trx system suppresses oxidative stress and counteracts DNA damage, lipid peroxidation, and protein carbonylation, thereby constraining downstream pathological events, including neuroinflammation, impaired neurogenesis, neurotransmitter imbalance, and ferroptosis. These pathological outputs, in turn, exacerbate oxidative stress and disrupt redox homeostasis, forming bidirectional vicious feedback loops that drive the progression of mental disorders.

### 3.1 Depression

Depression is a prevalent mental disorder that severely compromises physical and psychological well-being, with core manifestations of persistent low mood, anhedonia, and cognitive dysfunction (Sit et al., 2023). Authoritative statistics estimate that depression affects approximately 300 million individuals worldwide (Wang et al., 2025). Its pathogenic mechanisms encompass heightened neuroinflammation, impaired neurogenesis, and neurotransmitter dysregulation. Accumulating evidence has documented aberrant Trx-system expression in multiple animal depression models as well as human clinical samples.

Neuroinflammation is the CNS innate immune response to harmful stimuli, and it is well established as a key driver of depressive-like behaviors (Chen H. et al., 2025). Pre-clinical studies have detected markedly elevated inflammatory markers, including interleukin-1β (IL-1β) (Li et al., 2023), interleukin-6 (IL-6), and tumor necrosis factor-α (TNF-α) (Jang et al., 2021), in peripheral blood and cerebrospinal fluid of depressed patients relative to healthy controls. These elevated levels are positively correlated with disease severity (Li et al., 2024). Furthermore, patients with depression exhibit prominent differences from healthy controls with respect to microglial morphology, cytokine production, and activation status. Microglia are the resident innate-immune cells of the CNS. Upon activation by stressors or injuries, microglia undergo morphological transformation into amoeboid shapes and proliferate in response to inflammatory cues (Covelo and Araque, 2018). In depressive models, microglia are predominantly polarized toward the M1 phenotype, which drives excessive neuroinflammation and triggers depressive-like behaviors. By contrast, M2-polarized microglia facilitate neuronal repair and regeneration and exert neuroprotective functions (Kokkosis et al., 2024). Nevertheless, recent single-cell sequencing technologies have challenged this binary classification, as microglial activation exists along a continuous phenotypic spectrum with diverse molecular markers and receptor repertoires (Wang Q. Q. et al., 2026). In chronic stress and depression models, single-cell RNA-sequencing (scRNA-seq) studies have also uncovered complex microglial transcriptional changes that cannot be assigned to predefined phenotypes, highlighting the need to understand dynamic microglial state transitions (Böttcher et al., 2020; Samuels et al., 2023). Post-mortem analyses show increased mRNA expression of markers such as *CX3CR1* and *TMEM119* in brain regions including the inferior frontal gyrus, superior temporal gyrus, thalamus, and subventricular zone of patients with depression (Snijders et al., 2021). Studies have also reported NLRP3 inflammasome activation in blood cells from patients with major depressive disorder (MDD), and elevated serum IL-1β and IL-18 levels are positively correlated with Beck Depression Inventory (BDI) scores (Alcocer-Gómez et al., 2014). In mice subjected to chronic unpredictable mild stress (CUMS), hippocampal NF-κB expression is upregulated (Abd El-Fattah et al., 2018). *NLRP3* knockout blunts the NF-κB signaling pathway and alleviates depressive phenotypes (Su et al., 2017b).

Activation of the microglial NLRP3 inflammasome mediates the emergence of depressive-like behaviors by triggering neuroinflammation (Alcocer-Gómez et al., 2014). Antidepressant drugs such as fluoxetine (a selective serotonin reuptake inhibitor) ameliorate depressive-like behaviors by suppressing the secretion of pro-inflammatory cytokines from microglia and promoting M2-type polarization. Recombinant Trx-1 protein can also promote microglial polarization toward the M2 phenotype via inhibition of the mitogen-activated protein kinase (MAPK) signaling (Zhang X. et al., 2026). TXNIP expression is increased in the brains of mice exposed to chronic stress (Zhou et al., 2019). *In vitro* loss-of-function experiments show that *TXNIP* knockdown in BV2 microglia markedly attenuates lipopolysaccharide (LPS)-induced NLRP3 inflammasome activation and reduces pro-inflammatory cytokine release (Shi et al., 2026). Perilla aldehyde alleviates chronic stress-induced depressive-like behaviors by modulating the TXNIP/Trx-1/NLRP3 pathway (Song et al., 2018). Predictable chronic mild stress can suppress TXNIP expression and facilitate recovery from LPS-induced depressive-like phenotypes (Dang et al., 2019). In addition, excessive neuroinflammation impairs adult hippocampal neurogenesis and disturbs neurotransmitter homeostasis (Guo et al., 2024). Conversely, impaired neurogenesis and aberrant neurotransmitter signaling further aggravate neuroinflammation, forming a mutually reinforcing pathological feedback loop.

Neurogenesis refers to the process by which neural stem cells (NSCs) proliferate and differentiate into functional neurons, compensating for neuronal loss and supporting neuronal repair (Ribeiro and Xapelli, 2021; Zhao et al., 2024). Chronic stress impairs neural plasticity in the brain and particularly suppresses adult hippocampal neurogenesis (Jung et al., 2020). Hippocampal neurogenesis occurs predominantly in the subgranular zone of the dentate gyrus (DG) and persists throughout the lifespan (Gage, 2019). Clinical imaging studies have shown that patients with depression exhibit smaller hippocampal volumes compared with healthy controls (Gorham et al., 2019), and the degree of hippocampal atrophy is positively correlated with the disease duration of MDD (Boldrini et al., 2013). In patients with depression, both the abundance and differentiation capacity of hippocampal neural stem cells are markedly reduced, leading to neurological dysfunction as well as abnormalities in emotional and cognitive functions (Borsini et al., 2022). In addition, impaired angiogenesis within the DG of depressed patients is also associated with compromised neurogenesis (Wu et al., 2023). Preclinical studies have shown that mice subjected to mild restraint stress exhibit neuronal loss and dendritic atrophy in the CA1 region, accompanied by reduced DG neurogenesis (Du Preez et al., 2021). These findings suggest that impaired neurogenesis may constitute one key contributor to the pathogenesis of depression.

Fluoxetine may alleviate depressive symptoms by boosting neurogenesis (Zuo et al., 2024). Electroacupuncture treatment can promote brain-derived neurotrophic factor (BDNF)-dependent neurogenesis to ameliorate depressive behaviors induced by CUMS (Zhang L. et al., 2026). Nerve growth factor (NGF) is a key mediator facilitating neural regeneration (Bliźniewska-Kowalska et al., 2021). As an essential cofactor in NGF signaling, Trx-1 is significantly upregulated during NGF-triggered neuronal differentiation (Bai et al., 2003). Inhibition of Trx-1 diminishes dendritic outgrowth in primary cultured mouse cortical neurons and lowers the activation level of cAMP response element-binding protein (CREB) (Alejandra Llanes-Cuesta et al., 2024). CREB is a core transcription factor that governs the expression of multiple genes implicated in neuronal differentiation and synaptic plasticity (Landeira et al., 2018). Exogenously administered recombinant human Trx-1 can also boost DG neurogenesis and improve cognitive function in cerebral ischemia mice (Tian et al., 2014), implying that Trx-1 plays an important regulatory role in neurogenesis. Regular exercise upregulates Trx-1 expression to improve hippocampal neurogenesis and relieve chronic depressive-like phenotypes, an effect mediated by the β2-adrenoceptor-dependent extracellular regulatory kinase-1/2 (ERK-1/2)-β-catenin/Tcf signaling pathway (Kim and Leem, 2019). This observation confirms that Trx-1 mediated regulation of hippocampal neurogenesis can ameliorate depressive symptoms. Furthermore, *TXNIP* knockdown within the medial prefrontal cortex (mPFC) can reverse corticosterone (CORT) induced dendritic atrophy and prevent CORT-evoked depressive-like behaviors (Llanes-Cuesta et al., 2025).

Neurotransmitters are endogenous chemical messengers widely deployed in the CNS for intercellular signal transmission. Anhedonia, one of the core symptoms of depression, is closely associated with dopaminergic neuronal dysfunction (Cardona-Acosta and Bolanos-Guzman, 2023). Chronic stress suppresses dopaminergic neuron activity in the brain (Chang and Grace, 2014). Re-activating the firing of dopaminergic neurons can reverse depressive behavior in depressive-like rats (Tye et al., 2013; Friedman et al., 2014). Genetic variants of the D2 receptor also increase individual vulnerability to depression (Zhang et al., 2023). Loganin, an active component derived from traditional Chinese medicine, ameliorates CUMS-induced depressive behaviors by elevating dopamine (DA) levels(Guo et al., 2023). Accordingly, enhancing dopamine synthesis and release may contribute to the rapid relief of depressive symptoms. Another approved antidepressant, ketamine, exerts rapid antidepressant effects by blocking the burst-firing activity of N-methyl-D-aspartate (NMDA)-dependent lateral habenula (LHb) neurons, thereby disinhibiting downstream monoaminergic reward centers and rapidly improving mood (Kim et al., 2018; Yang et al., 2018). Serotonin (Mahar et al., 2014) and γ-aminobutyric acid (GABA) (Murrough et al., 2017) also play crucial roles in mood regulation, and their levels are markedly abnormal in patients with depression. BDNF is a pivotal neurotrophic factor. Its expression is reduced in the hippocampus and prefrontal cortex (PFC) of depressed individuals and declines further as disease progresses (Duman and Voleti, 2012). Exogenous BDNF administration produces prominent antidepressant effects (Shirayama et al., 2002), whereas dysfunction of the tropomyosin receptor kinase type B (TrkB) signaling blunts the therapeutic efficacy of antidepressant drugs (Monteggia et al., 2004).

Intriguingly, we have confirmed that Trx-1 can upregulate the expression of the key enzyme tyrosine hydroxylase (TH) in dopamine synthesis and promote DA release (Jia et al., 2015). DA agonists represent potential therapeutic agents for treatment-resistant depression. Furthermore, Trx-1 has also been reported to activate dopamine D1 receptor (D1R) and the NR2B/ERK-1/2-CREB pathway (Zhang X. et al., 2018). These findings indicate that Trx-1 modulates dopaminergic system function. Compared with WT mice, Trx-1-transgenic mice exhibit markedly elevated brain levels of DA and GABA (Huang et al., 2018b; Li et al., 2018). Long-term antidepressant treatment significantly increases the concentrations of serotonin and norepinephrine in the synaptic cleft (Connolly and Thase, 2012), alongside increased protein levels of Trx-1 and TrxR1 (Bharti et al., 2020). Trx-1 also serves as a critical mediator for BDNF-driven synaptic protein expression. The Trx-1 inducer geranylgeranylacetone (GGA) can substantially increase BDNF levels in the brain (Guo et al., 2022).

Collectively, these findings indicate that the Trx-1 system may play an important role in depression by modulating neuroinflammation, neurogenesis, neurotransmitter homeostasis, and other pathways. Nevertheless, its role in treatment-resistant depression remains poorly defined (Aydın et al., 2018). In addition, most evidence originates from preclinical observations, and human interventional data are scarce.

### 3.2 SCZ

SCZ is a highly heritable severe psychiatric disorder with a global lifetime prevalence of approximately 1%, which severely impairs thinking, perception, social interaction, and cognitive performance. Serum Trx-1 levels are elevated in both first-episode (Zhang et al., 2009) and chronic patients with SCZ (Owe-Larsson et al., 2011), and serum Trx-1 levels are positively correlated with positive psychotic symptoms of SCZ (Zhang et al., 2013). This suggests that Trx-1 may play an important role in the pathogenesis and psychopathology of SCZ. Unlike first-episode patients, chronic patients receiving long-term antipsychotic treatment show no significant differences in Trx-1 levels compared with healthy controls, and their Trx-1 levels are markedly lower than those in acute-phase patients. This implies that antipsychotic treatment may reduce Trx-1 levels, or may reflect alleviated OS in the chronic phase (Bas et al., 2017). Furthermore, Trx-1 levels are significantly negatively correlated with the Repeatable Battery for the Assessment of Neuropsychological Status (RBANS) attention index in patients with SCZ. In contrast, they show a significant positive correlation in healthy controls (Zhang et al., 2013). This phenomenon may be explained as follows: higher Trx-1 levels in healthy individuals confer better protection against oxidative damage, thereby improving brain function and cognitive performance, particularly attention. By contrast, under pathological OS, patients with SCZ may fail to produce sufficient Trx-1, leading to brain damage and cognitive decline. Cognitive impairment is a prominent feature of SCZ and is closely associated with reduced NMDAR function (Kruse and Bustillo, 2022). Animal models of SCZ show reduced NR2B expression, and restoring NR2B expression can improve cognitive function (Li et al., 2022). Accumulating evidence indicates that Trx-1 alleviates cognitive deficits in multiple disease models via the NR2B/ERK/CREB signaling pathway (Zhang X. et al., 2018; Qiu and Liu, 2022). Upregulation of Trx-1 and TrxR1 can mitigate neuronal injury and cognitive impairment in vascular dementia rats (Zhu et al., 2018). These findings suggest that modulating Trx-1 expression may improve cognitive dysfunction in patients with SCZ. Substantial evidence demonstrates dysfunction in GABA synthesis and transmission among patients with SCZ (Uliana et al., 2024). Compared with healthy controls, patients with SCZ exhibit reduced GABA levels in the nucleus accumbens (NAc) and thalamus. Trx-1 can enhance GABA biosynthesis.

Microdeletions at the 22q11.2 locus harboring the *TXNRD2* gene (encoding mitochondrial TrxR2) represent an important genetic risk factor for schizophrenia. TXNRD2 participates in ROS detoxification and protects mitochondria from oxidative damage by maintaining the pool of reduced Trx-2 (Lee et al., 2020). Impaired TXNRD2 function is linked to oxidative damage and abnormal energy metabolism, which further disrupt neuronal activity and connectivity, and is associated with SCZ (Kolar et al., 2023).

In addition, recent studies have identified *TXNIP* as a novel candidate gene for SCZ (Su Y. et al., 2017). DNA microarray analyses show that *TXNIP* resides on chromosome 1q21.1, a susceptibility locus for SCZ identified by genome-wide association studies (GWAS) (Brzustowicz et al., 2000). A study conducted in a Chinese population reported aberrant TXNIP expression in peripheral blood lymphocytes of SCZ patients (Cui et al., 2004). Another controlled clinical study found markedly elevated plasma TXNIP levels in first-episode and early-onset SCZ patients (Xue et al., 2022). Patients with higher TXNIP levels presented with more severe psychiatric symptoms relative to those with lower levels. Increased TXNIP expression accompanied by augmented OS was also detected in the PFC of MK-801-induced SCZ-like mouse models (Xue et al., 2022). Given the consistent observation of Trx-system perturbations in SCZ, targeting this pathway may offer potential as an adjuvant therapeutic strategy.

Nevertheless, alterations in peripheral blood TXNIP/Trx-1 show inconsistent results across independent patient cohorts. Such discrepancies may stem from differences in medication status, disease stage, and sample size across clinical cohorts. Causal evidence directly linking Trx system alterations to SCZ pathogenesis remains limited.

### 3.3 ASD

ASD is a neurodevelopmental disorder with onset in childhood or adolescence, characterized by social impairments, stereotyped behaviors, and cognitive dysfunction (Tong et al., 2024). Its pathogenesis involves OS, immune dysregulation, neuroinflammation, mitochondrial dysfunction, ferroptosis, and other processes (Rossignol and Frye, 2012). Clinical data show elevated serum Trx-1 levels in children with ASD (Al-Gayyar et al., 2011), which may serve as a novel independent diagnostic indicator for ASD. Serum Trx-1 expression levels are also positively correlated with disease severity (Zhang Q. et al., 2015; Abd-Allah et al., 2020).

*Shank3* is a well-recognized ASD susceptibility gene (Amal et al., 2020) that encodes a scaffold protein involved in dendritic spine morphology and synaptic transmission (Wan et al., 2021). Recent pre-clinical investigations confirm significantly reduced Trx-1 and TrxR1 activity in the brains of *Shank3* knockout ASD model mice (Bazbaz et al., 2024). *Shank3* mutations lead to excessive nitric oxide (NO) production, nitrosative stress, and aberrant protein S-nitrosylation (SNO) (Amal et al., 2020; Kartawy et al., 2021). Excess NO rapidly reacts with ROS to generate compounds such as peroxynitrite (ONOO^−^). These compounds then induce nitration of protein tyrosine residues or free tyrosine to produce 3-nitrotyrosine (Abdel-Haq et al., 2023), ultimately resulting in neuronal injury. Inhibition of Trx-1 increases 3-nitrotyrosine levels (Bazbaz et al., 2024). Trx-1 and its oxidoreductase modulate nitrosative stress via protein reduction and denitrosylation (Thom et al., 2012). Furthermore, Trx-1 can regulate NO production by denitrosylating neuronal nitric oxide synthase (nNOS) and inducing inducible nitric oxide synthase (iNOS) (Qu et al., 2012). These findings highlight a central role for the Trx-1 system in regulating oxidative-nitrosative stress. Notably, diminished Trx-1 activity following *Shank3* deletion partially arises from enhanced SNO triggered by excess NO (Bazbaz et al., 2024). Conversely, loss of *Shank3* reduces Ser40 phosphorylation, thereby suppressing Nrf2 nuclear translocation and consequently downregulating the expression of Trx-1 system proteins (Bjørklund et al., 2020; Nishimura et al., 2021). Increased OS and nitrosative stress caused by Trx-1 inhibition can also lead to synaptic defects (Abdel-Haq et al., 2023; Tripathi et al., 2023; Tripathi et al., 2024).

*Shank3* knockout (KO) mice show dysfunction in glutamatergic and GABAergic systems (Tripathi et al., 2023), which may be associated with reduced Trx-1. Pharmacological inhibition of the Trx-1 system in WT mice markedly augments nitrosative stress and synaptic deficits (Tripathi et al., 2023; Tripathi et al., 2024). In addition, oxidative stress may trigger synaptic damage and neuronal death, while elevated S-nitrosylation of synaptic proteins can also contribute to synaptic impairment (Hamoudi et al., 2021; Kartawy et al., 2021).

Mitochondria are the major cellular source of ROS and a primary target of oxidative injury. Compelling evidence points to mitochondrial dysfunction, arising from excessive ROS production and/or insufficient antioxidant defense, in peripheral tissues and brain samples from ASD patients (Chełchowska et al., 2025). Nevertheless, mitochondrial dysfunction is only observed in a subset of ASD patients. Approximately 4% of children present with classically defined mitochondrial disease, reflecting substantial heterogeneity of mitochondrial involvement across ASD cohorts (Frye, 2020). With massive intracellular ROS accumulation, TXNIP translocates into mitochondria via incompletely defined mechanisms, binds to the disulfide bond of Trx-2, and inhibits Trx-2’s reductive function, leading to the formation of the TXNIP/Trx-2 complex. The TXNIP/Trx-2 complex boosts mitochondrial ROS generation and induces ASK1 phosphorylation, thereby promoting mitochondrial-mediated apoptosis. TXNIP also induces mitochondrial dysfunction by promoting mitochondrial membrane depolarization, increasing ROS production, and impairing ATP synthesis (Kim et al., 2023). Moreover, TXNIP and Trx regulate peroxisome proliferator-activated receptor α (PPARα), a key modulator of mitochondrial respiration and biogenesis. TXNIP may exert its mitochondrial effects through Trx-mediated regulation of PPARα (Yoshioka and Lee, 2014). In addition, studies suggest that aberrant SNO signaling induced by *Shank3* mutations may mechanistically explain mitochondrial dysfunction in ASD (Kartawy et al., 2021).

Patients with ASD also frequently exhibit abnormal inflammatory responses and immune system dysregulation (Avolio et al., 2026). ST36 acupuncture alleviates behavioral deficits in autistic rats by suppressing TXNIP-mediated NLRP3 activation (Zhao et al., 2022). The Trx-1/TrxR1 system is also expressed in immune cells and is critical for normal immune-cell proliferation. Furthermore, the Trx-1/TrxR1 redox system regulates apoptosis through multiple mechanisms. Therefore, Trx-1/TrxR1 expression and activity may be required for immune cell proliferation and anti-apoptotic effects (Muri and Kopf, 2021). Clinical investigations have documented perturbed Trx-1/TrxR1 redox balance in T-cells and neutrophils isolated from ASD patients (Alshehri et al., 2023a; Alshehri et al., 2023b).

Genetic-screening studies have identified aberrant expression of ferroptosis-related genes in children with ASD (Wu X. et al., 2022). Glutathione peroxidase 4 (GPx-4) is a GSH-dependent canonical antioxidant enzyme and is regarded as a key endogenous inhibitor and biomarker of ferroptosis. In ASD model mice, GPx-4 expression is significantly decreased, and upregulating GPx-4 can ameliorate social behavioral deficits and repetitive stereotyped behaviors (Wu H. et al., 2022; Luo et al., 2023). The Trx-1 system is a critical defense mechanism against ferroptosis (Wang W. et al., 2026). It indirectly maintains the cellular reductive environment required for optimal GPx-4 activity. Trx-1 induces GSH synthesis (Zaobornyj et al., 2019), and the Trx-1 system can partially compensate for GSH depletion (Du et al., 2013). Thus, Trx-1 and GSH cooperatively maintain redox homeostasis. Curdione suppresses ferroptosis by modulating the Keap1/Trx-1/GPx-4 signaling pathway (Wang et al., 2023). In Parkinson’s disease (PD) models, Trx-1 also confers protection against ferroptosis by regulating GPx-4 activity and GSH levels (Bai et al., 2021). Recently, TXNIP has been recognized as a novel positive regulator of ferroptosis. Activation of the hippocampal neuronal TXNIP/Trx-1/GPx-4 signaling cascade promotes ferroptosis (Zhang et al., 2022). Curcumin exerts protective effects by inhibiting TXNIP/Trx-1/GPx-4-mediated ferroptosis (Chen et al., 2024). Taken together, the Trx system may ameliorate ASD-like phenotypes through multiple distinct pathways (Figure 4).

**FIGURE 4:**
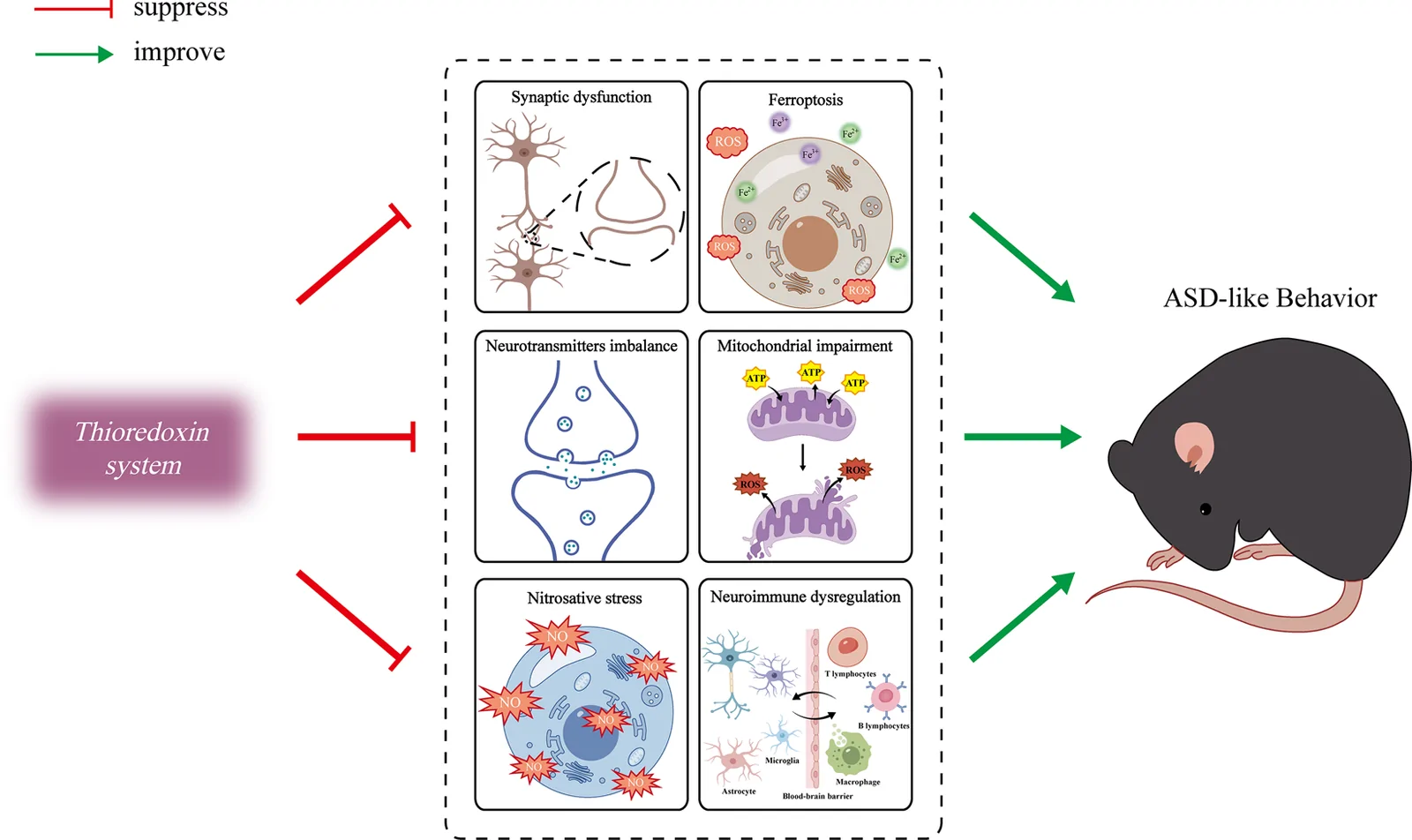
The thioredoxin system improves the ASD-like behaviors in mice by regulating synaptic dysfunction, ferroptosis, neurotransmitter imbalance, mitochondrial damage, nitrosative stress, and neuroimmune dysregulation.

Biomarker findings for the Trx system in ASD show substantial heterogeneity. *Shank3* mutations account for only a small proportion of human ASD cases, which may limit the generalizability of relevant conclusions. In addition, existing cross-sectional studies cannot distinguish whether peripheral Trx-1 alterations represent primary pathogenic drivers or secondary compensatory responses induced by neurodevelopmental stress, immune activation, or metabolic disturbance. Future, multi-center, large-sample cohort studies are warranted.

### 3.4 SUD

SUD is a catastrophic brain disease and a global public health crisis. Addictive drug abuse triggers excessive OS burden within the brain reward circuit (Skrabalova et al., 2013). Previous reports have indicated that NO-dependent guanylate cyclase activation and elevated cyclic guanosine monophosphate (cGMP) signaling are associated with morphine tolerance and dependence (Hussain et al., 2024). Chronic morphine exposure suppresses antioxidant enzyme activity, including superoxide dismutase (SOD) and GSH, and disrupts cerebral redox homeostasis (Hejnova et al., 2024). Methamphetamine (METH) exposure promotes excessive DA release from midbrain dopaminergic nerve terminals and competitively binds to DA transporters, thereby reducing dopamine reuptake. Spontaneous oxidation of excess DA generates abundant free radicals and oxidative metabolites, resulting in severe neuronal injury (Huang et al., 2013). METH abuse also elevates cerebral glutaraldehyde levels and reduces GSH content (Jang et al., 2017). Cocaine also increases ROS levels and impairs antioxidant defense systems in the brain reward circuit. Antioxidant agents can alleviate drug-induced oxidative damage and suppress drug dependence and behavioral sensitization (Asadi Akbarabadi et al., 2021; Kamiński et al., 2024; Katebi et al., 2024).

Studies have demonstrated aberrant Trx-1 expression in multiple brain regions of mice treated with morphine and METH (Luo et al., 2012; Huang et al., 2018b). Overexpression of Trx-1 blocks the expression of morphine- and METH-induced conditioned place preference (CPP) by regulating DA and GABA. GGA not only protects the liver and kidneys against morphine-induced injury but also inhibits the reinstatement of morphine-CPP by modulating the NR2B/ERK pathway in the NAc and hippocampus (Guo et al., 2018). In contrast, Trx-1 downregulation facilitates METH-CPP reinstatement (Huang et al., 2018c). These findings suggest that the Trx-1 system may serve as a promising therapeutic target for SUD.

Nevertheless, most evidence in this field originates from rodent CPP models. Although the CPP paradigm can assess drug reward and motivational properties in rodents, it measures relatively simple associative learning behaviors. It cannot fully recapitulate the multifactorial complexity of human SUD (Corley et al., 2024). Accordingly, translational extrapolation from rodent CPP findings to human SUD should be interpreted cautiously. In addition, the high comorbidity of psychiatric and somatic disorders among individuals with substance use is a non-negligible confounding factor.

### 3.5 Common and distinct mechanistic features in mental disorders

Despite phenotypic differences among these four psychiatric disorders, they share convergent molecular cascades. Shared mechanisms: Oxidative-nitrosative stress burden acts as a unified upstream trigger that promotes TXNIP upregulation (Ogata et al., 2013). TXNIP-NLRP3 inflammasome activation mediates neuroinflammatory responses across disease contexts (Tsubaki et al., 2020). Impairment of the Nrf2-ARE antioxidant transcriptional pathway is frequently reported (Bhandari et al., 2021). Nrf2 regulates the transcription of antioxidant genes belonging to the Trx system and GSH system. They also share overlapping downstream pathologies (Ferrando et al., 2025). Disease-specific features: Differential Trx-1 expression. For instance, Trx-1 expression is reduced in the hippocampus of depression (Song et al., 2018), whereas plasma Trx-1 levels are elevated in SCZ (Zhang et al., 2009). Chronic stress may deplete antioxidant reserves and reduce Trx system function (Llanes-Cuesta et al., 2025). By contrast, elevated Trx-1 in SCZ may reflect a compensatory antioxidant response (Cecerska-Heryć et al., 2024). GWAS has identified *TXNIP* as a susceptibility locus for SCZ (Su Y. et al., 2017), yet it has not been established as a major genetic risk gene in other psychiatric disorders. Final phenotypes: mood disturbances (depression), psychosis and cognitive deficits (SCZ), social impairments and stereotyped behaviors (ASD), and compulsive drug-seeking behavior (SUD).

## 4 Conclusion and perspectives

Psychiatric disorders exert profound adverse effects on physical and mental health. Their pathogenesis arises from complex interactions between genetic susceptibility and environmental risk factors, and effective therapeutic options remain limited. Substantial evidence indicates that the Trx system serves as a key homeostatic signaling hub within the CNS and helps regulate multiple pathological mechanisms underlying psychiatric diseases. However, current studies still have notable limitations. First, most mechanistic evidence derives from animal experiments and small-sample human observational cohorts (Zhang Q. et al., 2015; Guo et al., 2018). Large-sample and cross-diagnostic comparative studies in clinical populations remain scarce. It remains challenging to distinguish whether Trx-system perturbations represent primary pathological alterations or secondary downstream consequences of disease. Second, animal disease models cannot faithfully recapitulate the slow, multifactorial pathological processes and clinical phenotypes of human patients. Psychiatric disorders exhibit clear epidemiological sex dimorphism, with the prevalence of depression in females approximately 1.5-fold higher than in males (World Health Organization, 2025b). Nevertheless, most preclinical studies exclusively employ male experimental animals. There is a lack of research investigating whether sex-hormone-mediated differential regulation of the Trx system modulates disease susceptibility, disease progression, and therapeutic responses in psychiatric disorders. Third, although plasma Trx-1 and TXNIP hold potential as biomarkers, their sensitivity and specificity need further rigorous verification. Peripheral factors such as medication status, inflammation, and age can substantially alter circulating concentrations, and these peripheral markers may not faithfully reflect CNS molecular levels. Furthermore, multiple human cohort studies have yielded inconsistent or invalid results, which may be affected by factors such as sample size and patient subtype heterogeneity.

### 4.1 Therapeutic prospects and translational bottlenecks

Multiple pharmacological agents that regulate the Trx system, including GGA (Guo et al., 2018), resveratrol (Farkhakfar et al., 2023; Zeng et al., 2024), Panax notoginseng saponins (Zeng et al., 2014), alongside other bioactive small-molecule compounds, have shown neuroprotective effects in rodent disease models. The clinical translation of Trx-targeted strategies for psychiatric disorders remains hindered by multiple practical bottlenecks. First, specific Trx-1 inducers with favorable blood-brain barrier (BBB) permeability are lacking. Second, Trx and GSH antioxidant networks show partial functional redundancy. Interventions targeting the Trx system may trigger compensatory upregulation of the GSH pathway and blunt expected therapeutic effects. Third, given that the TXNIP/Trx axis fulfills critical redox functions across nearly all peripheral tissues, non-selective systemic modulation carries potential safety risks. Furthermore, no completed randomized controlled human trials have assessed the effects of Trx system modulators in psychiatric disorders.

### 4.2 Priorities for future research

Future studies should further elucidate the upstream molecular cascades that regulate Trx-1 system activity under pathological stress associated with psychiatric disorders. Additional experiments in female animals are needed to dissect the crosstalk between sex hormones and redox signaling. High-throughput drug-screening technologies should be deployed to identify novel small-molecule Trx-system modulators featuring desirable BBB permeability and cell-type selectivity. The efficacy and safety of these candidate compounds need rigorous validation using standardized preclinical research paradigms. Furthermore, combinatorial therapeutic strategies targeting the Trx system alongside other redox- and inflammation-related signaling nodes should be explored to overcome the limitations of single-target interventions.

In summary, accumulating evidence highlights the TXNIP/Trx axis as a promising pre-clinical target for deciphering redox-linked disease mechanisms across multiple psychiatric conditions. Given the substantial gaps separating preclinical observations from real-world human clinical realities, concerted, multifaceted mechanistic, biomarker, and translational research efforts are required to advance Trx system-modulating interventions from basic research toward practical psychiatric clinical applications.
